# Dyspeptic Correspondence

**Published:** 1858-05

**Authors:** 


### DYSPEPTIC CORRESPONDENCE.

DEAR DOCTOR: Do get your friend, the Minister, to give Dyspeptics an account of the *trotting-match* between "nimble stomach" and "lazy liver."—I should like very much to read it, as it would doubtless suggest many useful ideas to myself and others, who are laboring under that awfully mean disease—the worse for being brought on by over-eating, and want of exercise, and too much care and anxiety about what we shall eat, what we shall drink, and wherewith all shall we be clothed?

I was just leaving my office to go see the doctor, and tell him I felt awfully bad, and to ask him for some other kind of medicine. I dropped in at my brother's, he keeps all the periodicals, and among them "HALL'S JOURNAL," of which I am a fond reader; picked up the April number; thought I would go back to the office to see what was in it; read through Throat-Ail; was so much pleased with the Minister's letter, thought I would write at once to you, and get you to get the Minister to let us have his experience. Though an entire stranger to you, I

think from the way you treat Alice and Bob, you will not refuse to gratify a host of poor dyspeptic creatures, who (something like the fox, and not exactly like him), *too*, have to say sour grapes to sweet candies, not because they *can't* but because they *daren't* eat 'em.

I have four Bobs and Alices—not exactly that, but one Bob and three Alices—that I will set up against any other set of Bobs and Alices either for eating candy, romping, or anything else: should be glad to have yours with them.

Hope you will excuse the liberty, Doctor; but I just felt in the humor of writing you, to get a description of that "race."

